# Transmission of *Mycobacterium chimaera* from Heater–Cooler Units during Cardiac Surgery despite an Ultraclean Air Ventilation System

**DOI:** 10.3201/eid2206.160045

**Published:** 2016-06

**Authors:** Rami Sommerstein, Christian Rüegg, Philipp Kohler, Guido Bloemberg, Stefan P. Kuster, Hugo Sax

**Affiliations:** University Hospital of Bern, Bern, Switzerland (R. Sommerstein);; University Hospital of Zurich, Zurich, Switzerland (R. Sommerstein, C. Rüegg, P. Kohler, S.P. Kuster, H. Sax);; University of Zurich, Zurich (G. Bloemberg)

**Keywords:** Mycobacterium chimaera, bacteria, aerosols, tuberculosis and other mycobacteria, heater–cooler units, surgical wound infection, cardiac surgical procedures, nontuberculous mycobacteria, cross infection, disease outbreaks, airborne transmission pathway

## Abstract

Heater–cooler units (HCUs) were recently identified as a source of *Mycobacterium chimaera* causing surgical site infections. We investigated transmission of this bacterium from HCUs to the surgical field by using a thermic anemometer and particle counter, videotape of an operating room equipped with an ultraclean laminar airflow ventilation system, and bacterial culture sedimentation plates in a nonventilated room. Smoke from the HCU reached the surgical field in 23 s by merging with ultraclean air. The HCU produced on average 5.2, 139, and 14.8 particles/min in the surgical field at positions Off, On/oriented toward, and On/oriented away, respectively. Culture plates were positive for *M. chimaera* <5 m from the HCU in the test room. These experiments confirm airborne transmission of *M. chimaera* aerosols from a contaminated HCU to an open surgical field despite ultraclean air ventilation. Efforts to mitigate infectious risks during surgery should consider contamination from water sources and airflow-generating devices.

## Abstract 2

All such units should be separated from air that can gain access to sterile areas.

Several independent studies have reported postoperative prosthetic-valve endocarditis caused by waterborne bacteria, such as *Legionella* spp. and *Mycobacterium* spp. (*1*–*5*). The mode of transmission was not fully established until airborne transmission of *Mycobacterium chimaera* from heater–cooler units was reported (*1*,*6*). These units are widely used in open-chest heart surgery as an essential part of extracorporeal circulation but have been suggested as being a risk for infection (*7*).

Ultraclean air ventilation systems produce a vertically directed flow of filtered ultraclean air from the ceiling to the sterile operating area, also known as laminar airflow. The protective advantage of these systems compared with whole-room, turbulent, clean air ventilation has been questioned. Although Bosanquet et al. suggested that laminar airflow is superior in reducing surgical site infections in vascular patients (*8*), Brandt et al. reported an increase in surgical site infections in orthopedic surgery after use of laminar airflow systems (*9*). The hazard of horizontal air flow disrupting laminar airflow has been studied for forced-air warming systems that prevent hypothermia (*10*–*12*). Although these devices contaminate ultra-clean air ventilation systems, no definite link to an increased risk for surgical site infection has been established (*10*–*12*).

The considerable horizontal airflow generated by heater–cooler units might disrupt vertical ultraclean airflow, which could be a potential mechanism for transmission of pathogens from a contaminated heater–cooler unit to a surgical site. Thus, we conducted a series of technical and microbiological experiments to investigate the potential airborne transmission pathway of pathogens, such as *M. chimaera*, from a contaminated heater–cooler unit to the surgical field.

## Methods

### Setting

Experiments were performed at the University Hospital of Zurich (Zurich, Switzerland) in 2014. This hospital is an 800-bed tertiary-care center.

### Heater–Cooler Units

**Figure 1:**
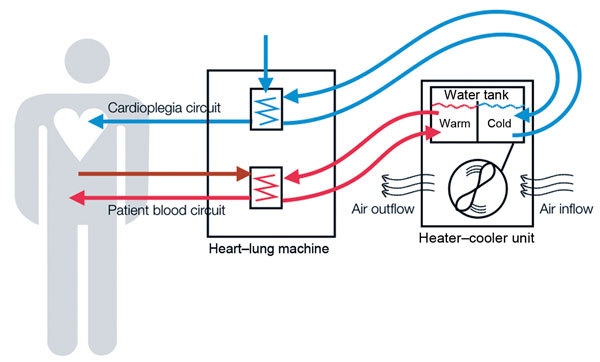
Schematic representation of heater–cooler circuits tested for transmission of *Mycobacterium chimaera* during cardiac surgery despite an ultraclean air ventilation system. Blue arrows indicate cold water flow, and red arrows indicate hot water flow and patient blood flow.

A single model of heater–cooler unit (Model 3T; Sorin, Milan, Italy) was used as a test unit for all experiments in this study. Heater–cooler units are stand-alone devices that contain a tank that holds filtered tap water, which serves as transfer fluid to control the temperature of patient blood and cardioplegia solution (Figure 1). The tank has 2 main compartments, 1 for warming water and 1 for cooling water. Water from the 2 tanks is continuously pumped through 2 circuits that consist of tubes and temperature exchange elements attached to a heart–lung machine. The warm water circuit controls blood temperature of the patients, and the cold water circuit cools the cardioplegia solution. Thus, water temperature in the tank might range between 2°C and 41°C during operation; water returns to room temperature on standby. The tank features filling and overflow tubes, heating and cooling coils, probes, and stirring devices. It is not airtight. The space beneath the tank holds a radiator to dissipate superfluous heat produced through water cooling. The efficacy of this radiator is increased by a fan that ensures a constant airflow through ventilation grid openings on either side of the heater–cooler unit housing.

For all experiments, the heater–cooler unit was preset to cooling of the cardioplegia circuit water to 4°C and the patient blood circuit water to 37°C to ensure maximal performance and ventilation when turned on.

### Cardiac Surgery Operating Room and Ventilation System

The cardiac surgery operating room used in this study is fully functional: floor surface area is 6 m × 7 m and height 3 m. The ultraclean air ventilation system in the operating room consists of a ceiling outlet (2 m × 2.4 m) equipped with a high-efficiency particle air filter (HEPA H14). At the annual quality control check on December 14, 2014, the airflow was 0.24 m/s at 0.2 m beneath the ceiling in the center of the outlet (check was performed according to standard 14644–3:2005 by the International Organization for Standardization) (*13*).

### Smoke Dispersal Experiments in a Functional Operating Room

On September 26, 2014, smoke dispersal experiments were performed in the surgical operating room in the presence of 3 researchers but no clinical staff or patient. The smoke source (Ventilax/Lüftax; AB Björnax, Strassa, Sweden) was placed at the vertical and horizontal center 0.2 m from the ventilation grid openings of the heater–cooler unit facing the operating table. Two test runs with opposite heater–cooler unit orientation were conducted, the first with ventilation airflow directed away from the operating table, and the second with ventilation airflow directed toward the operating table. The experiments were recorded by using 2 video cameras at eye level at a horizontal angle of 90°.

### Particle Counts in a Functional Operating Room

On December 11, 2014, a laser counter (Handilaz Mini; Particle Measuring Systems Inc., Boulder, CO, USA) was placed at the usual location of the open chest surgical field 2 m below the ultraclean air ventilation system ceiling outlet with the sampling opening pointing upwards. The heater–cooler unit was positioned at its usual location 2 m from the operating table and turned off to obtain background measurements. During a break in obtaining measurements, the heater–cooler unit was turned on and the airflow was oriented toward the operating table. After another short break in obtaining measurements, the airflow was oriented away from the operating table. The particle counter sampled air with a rate of 1.0 ft^3^/min for at least 4 min/configuration. Particle counts were reported overall and according to the 6 gates of the device that yield counts per particle size range.

### *M. chimaera* Sedimentation in a Test Room

On September 26, 2014, a heater–cooler unit with proven contamination of the water tank by *M. chimaera* (*6*) was used. The unit was positioned in the middle of a nonventilated office (area 4 m × 7 m, height 2.8 m). Sampling was performed by using passive air sedimentation on Middlebrook 7H11 agar culture plates (diameter 10 cm). Three plates were placed on the ground and positioned in a line 3 m, 4 m, and 5 m from the operating room heater–cooler unit, and the main ventilation airstream was directed toward the plates. A room of identical configuration 2 floors below was used as a control setting without a heater–cooler unit. Identical culture plates were placed at the same locations. Plates were left open for 4 hours and then sealed with parafilm, wrapped in a plastic bag to prevent desiccation, and immediately transported to a laboratory where they were incubated for 7 weeks at 37°C. Numbers of mycobacterial CFUs units were obtained, and species identification was performed by using 16S rRNA gene sequencing as described (*14*).

## Results

### Smoke Dispersal Experiments in a Functional Operating Room

**Figure 2:**
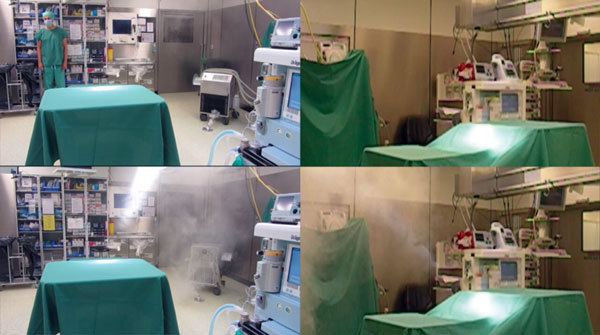
Video image captures showing effect of heater–cooler unit orientation on smoke dispersal in a cardiac surgery room and transmission of *Mycobacterium chimaera* during cardiac surgery despite an ultraclean air ventilation system. The device was switched on and began to ventilate 10 s after the start of the video. Frames on the left show an overview including unit placement. Frames on the right provide a lateral view of the operating field under the laminar airflow. Simultaneously recorded videos in the upper 2 frames show the first scenario, in which the main ventilation exhaust was directed *away* from the operating field. Simultaneously recorded videos in the lower 2 frames show the second scenario, in which the main ventilation exhaust was directed toward the operating field.

In the first scenario, in which the heater–cooler unit ventilation airflow was directed away from the operating field, the test smoke was aspirated by the heater–cooler unit through the ventilation grid, expelled through the ventilation grid on the opposite side, and evacuated by the ventilation exhaust opening in the wall of the operating room (Figure 2; Video). In the second scenario, in which the heater–cooler unit ventilation airflow was directed toward the operating field, smoke was blown away from the heater–cooler unit in a climbing airflow at an angle of 30°–60°. The smoke reached the ultraclean airflow 0.5 m beneath the ceiling outlet after 13 s, which resulted in a calculated mean airflow velocity of 0.23 m/s. The smoke then reached the surgical field 10 s later, which resulted in a calculated mean velocity of 0.15 m/s.

### Particle Counts in a Functional Operating Room

**Figure 3:**
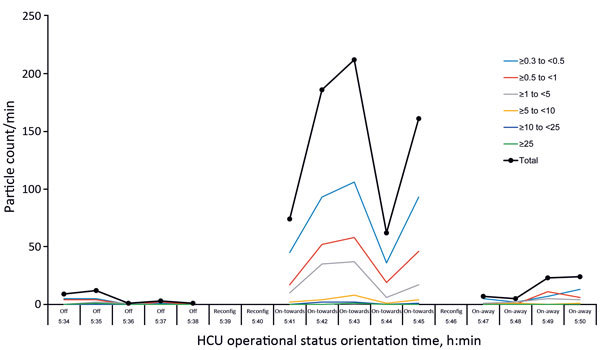
Laser particle measurements in cardiac operating room tested for transmission of *Mycobacterium chimaera* during surgery despite an ultraclean air ventilation system. Shown are measurements over time regarding heater–cooler unit (HCU) operational status (Off/On) and orientation (toward/away) with respect to the operating table. Lines indicate particle size ranges (in micrograms) captured by 6 gates and total particle count of the laser particle counter. Reconfig, time to reconfigure HCU status.

The laser particle counter captured 5.2 particles/min (range 1–12 particles/min) when the heater–cooler unit was turned off for 5 min (background measurement), 139 particles/min (range 62–212 particles/min) when the heater–cooler unit was turned on and its airflow oriented toward the operating table for 5 min, and 14.8 particles/min (range 5−24 particles/min) when the heater–cooler unit was turned on and its airflow was oriented away from the operating table for 4 min. Results are shown in Figure 3.

### *M. chimaera* Sedimentation in a Test Room

With the heater–cooler unit operating for the full 4 hours during which the plates were left open, the plate 3 m from the heater–cooler unit grew 2 CFUs of *M. chimaera* and the plate 5 m from the heater–cooler unit grew 1 CFU. The plate 4 m from the unit and all plates in the control test office without a unit remained sterile.

## Discussion

Our goal was to substantiate the recently hypothesized transmission pathway of potential pathogens (*1*,*6*) from a heater–cooler unit to the surgical field by an airborne route in a cardiac surgical operating room equipped with an ultraclean air ventilation system. Smoke dispersal experiments demonstrated that air originating from the heater–cooler unit was propelled by the ventilator of the unit to merge with the ultraclean airflow near the ceiling and ultimately reached the surgical field. This finding was corroborated by particle measurements in the same setup and showed increased particle counts in the surgical field when the heater–cooler unit airflow was oriented toward the operating table than at baseline and when the opposite heater–cooler unit orientation was used. Furthermore, we demonstrated that viable *M. chimaera* can be recovered on sedimentation plates placed in the air outflow path at a distance of up to 5 m from a contaminated heater–cooler unit. These results corroborate and extend previous observations (*6*). Previously, bacteria-positive air cultures had been demonstrated only when an active air sampling device was used.

These findings have major and novel implications in 3 areas. First, they substantiate the airborne transmission pathway for outbreaks of *M. chimaera* cardiac implant–related infections (*1*,*6*). Second, they provide prototypical evidence for the failure of ultraclean airflow ventilation systems in preventing surgical site infection. Third, they exemplify the potential infectious risks related to add-on airflow producing devices in the operating room.

A recent outbreak investigation of 6 case-patients with postoperative *M. chimaera* infections showed that *M. chimaera* contaminate heater–cooler unit water and are dispersed into the air (*6*). In that study, when heater–cooler units contaminated with *M. chimaera* were turned on, air cultures associated with the unit became contaminated with the same mycobacteria, which was proven in 1 instance to display the same random amplified polymorphic DNA PCR pattern. Conversely, air cultures remained bacteria negative when heater–cooler units were switched off or were running but not contaminated. Given the bacteria-positive air cultures and the absence of another common source of infection among the 6 patients, contamination of cardiac implants or the surgical field during surgical intervention had to be assumed. Similar to results of that report, contaminated heater–cooler units and associated infections have been reported in other hospitals in Europe and the United States (*1*,*6*,*15*,*16*).

Smoke dispersal experiments visualize the airflow pathway by which bacterial aerosols can access the surgical field, and particle count experiments showed a major increase in contamination of air reaching the surgical field. Furthermore, *M. chimaera* grew not only in samples of a considerable volume of air (*6*) but also on sedimentation plates up to 5 m from a heater–cooler unit in a nonventilated environment. Thus, the air concentration of mycobacteria was high enough to contaminate implant devices or the surgical field. The sedimentation culture experiments had to be conducted in a test setting because using a contaminated heater–cooler unit in the operating room was no longer advisable. In several other outbreaks of cardiac surgical site infections with nontuberculous mycobacteria, a transmission pathway by air was considered but not proven (*3*,*4*,*17*). Other members of the *M. avium* complex have been shown to aerosolize from water into air (*18*–*20*).

The association of ultraclean air ventilation systems with increased rates of surgical site infections in an epidemiologic evaluation (*9*) has sparked a debate on the benefit of such systems to prevent surgical site infection in a range of settings for which it has been shown to be effective (*8*,*21*–*23*). Thus, current guidelines offer no recommendation (unresolved issue) for performing orthopedic implant operations in rooms supplied with laminar airflow (*24*). The operating room smoke and particle count experiments in this study and recently described cases of *M. chimaera* infections in open-chest heart surgery patients (*1*,*6*) demonstrate a possible mechanism of how ultraclean air ventilation systems could fail to prevent, or even contribute to, surgical site infections. The design quality of ultraclean air ventilation systems has been reported as a major factor in their protective function (*25*). This design quality might also play a pivotal role in the transmission pathway of *M. chimaera*.

Our evidence also highlights the risks associated with devices that generate airflows in the operating room and that were not part of the original operating room air management design. Wood et al. (*10*) attempted to establish links between airflow-generating devices in operating rooms, such as forced-air warmers to maintain physiologic patient body temperature, and an increased rate of surgical site infections. However, this attempt was unsuccessful because results of microbial sampling could not be incriminated as a real risk for nosocomial infection (*11*) or because, although forced-air warmer blowers emitted airborne particles within the size range of free-floating bacteria and fungi, an association with surgical site infections was not investigated (*12*). A recent review reported that forced-air warmers contaminate ultraclean air ventilation systems (*10*). However, there appears to be no definite link to an increased risk for surgical site infections on the basis of current research (*10*). Although our experiments were not designed to answer the question of the overall benefit or harm of ultraclean air ventilation systems, the results exemplify that the combination of a contaminated horizontal airflow and laminar airflow can be responsible for surgical site infections.

Since 2014, heater–cooler units at the University Hospital of Zurich have been uniformly positioned away from the surgical field, and their exhaust air is now captured by a secondary housing and reliably channeled to the operating room exhaust. Although *M. chimaera* has regrown in some of the monthly water cultures from most factory-new heater–cooler units delivered in January 2014 after 1 year of operation on a daily water-change with filtered tap water scheme, air cultures in the exhaust air have consistently remained bacteria negative.

Our investigations have some limitations. First, the number of experiments was relatively small. However, results were unequivocal and consistent with previous findings (*1*,*6*). Second, the specific design of operating rooms, ventilation systems, and the heater–cooler unit model and the interplay of these elements affect the results and limit generalizations. However, specific design characteristics of the operating room ventilation system might potentially explain why infected patients were associated with only some of the many cardiac surgery centers in which heater–cooler units were found to be contaminated by *M. chimaera*. Third, our experiments were not designed to determine how nontuberculous mycobacteria left the heater–cooler unit water tank and reached the airflow and how the surplus of particles in the heater–cooler unit airflow was generated. These questions need further investigation, including other brands of heater–cooler units. Fourth, our experiments targeted only a nontuberculous mycobacterium that was found to have a major role in the etiology of surgical site infections. Thus, further testing of other pathogens that might be transmitted by the same route should be conducted. A recent study by Knibbs et al. showed that *Pseudomonas aeruginosa* aerosols emitted by coughing cystic fibrosis patients can become airborne, remain viable for 45 min, and travel up to 4 m (*26*).

In conclusion, results of our experiments substantiate the recently discovered airborne pathway of a pathogen from a contaminated heater–cooler unit to a surgical field. The specific role of ultraclean air ventilation systems in this pathway needs to be further investigated. Until more detailed evidence is available regarding this issue, all heater–cooler units should be reliably separated from air that can gain access to sterile areas and instruments, and devices that generate drafts should be banned from the operating room.
